# Impact of free maternity services on outcomes related to hypertensive disorders of pregnancy at Moi Teaching and Referral Hospital in Kenya: a retrospective analysis

**DOI:** 10.1186/s12884-023-05381-3

**Published:** 2023-02-06

**Authors:** Marie Buitendyk, Wycliffe Kosgei, Julie Thorne, Heather Millar, Joy Marsha Alera, Vincent Kibet, Christian Ochieng Bernard, Beth A. Payne, Caitlin Bernard, Astrid Christoffersen-Deb

**Affiliations:** 1 School of Medicine, Department of Obstetrics and Gynecology, University of Toronto, 27 King’s College Circle, Toronto, ON M5S Canada; 2 Moi Teaching and Referral Hospital, Eldoret, Kenya; 3 School of Medicine, Department of Obstetrics and Gynecology, Moi University, Eldoret, Kenya; 4 AMPATH (Academic Model Providing Access to Health Care) Kenya, P.O. Box 4606, Eldoret, Kenya; 5 School of Medicine, Department of Obstetrics and Gynecology, University of British Columbia, Vancouver, British Columbia V6T 1Z4 Canada; 6 School of Medicine, Department of Obstetrics and Gynecology, Indiana University, 107 S Indiana Ave, Bloomington, IN 47405 USA

**Keywords:** Hypertensive disorders of pregnancy, Preeclampsia, Eclampsia, Maternal mortality, Neonatal mortality, Free maternity care

## Abstract

**Background:**

Preeclampsia is a major contributor to maternal and neonatal mortality worldwide. Ninety-nine percent of these deaths occur in resource limited settings. One of the greatest barriers to women seeking medical attention remains the cost of care. Kenya implemented a nation-wide policy change in 2013, offering free inpatient maternity services to all women to address this concern. Here, we explore the impact of this policy change on maternal and neonatal outcomes specific to the hypertensive disorders of pregnancy.

**Methods:**

We conducted a retrospective cross-sectional chart review of patients discharged or deceased with a diagnosis of gestational hypertension, preeclampsia, eclampsia or HELLP syndrome at a tertiary referral center in western Kenya one year before (June 1, 2012-May 31, 2013) and one year after (June 1, 2013-May 31, 2014) free maternity services were introduced at public facilities across the country. Demographic information, obstetric history, medical history, details of the current pregnancy, diagnosis on admission and at discharge, antepartum treatment, maternal outcomes, and neonatal outcomes were collected and comparisons were made between the time points.

**Results:**

There were more in hospital births after policy change was introduced. The proportion of women diagnosed with a hypertensive disorder of pregnancy was higher in the year before free maternity care although there was a statistically significant increase in the proportion of women diagnosed with gestational hypertension after policy change. Among those diagnosed with hypertensive disorders, there was no difference in the proportion who developed obstetric or medical complications. Of concern, there was a statistically significant increase in the proportion of women dying as a result of their condition. There was a statistically significant increase in the use of magnesium sulfate for seizure prophylaxis. There was no overall difference in the use of anti-hypertensives between groups and no overall difference in the proportion of women who received dexamethasone for fetal lung maturity.

**Conclusions:**

Free maternity services, however necessary, are insufficient to improve maternal and neonatal outcomes related to the hypertensive disorders of pregnancy at a tertiary referral center in western Kenya. Multiple complementary strategies acting in unison are urgently needed.

## Background

Preeclampsia/eclampsia continues to be a leading contributor to global maternal and neonatal mortality [1]. Hemorrhage, sepsis and preeclampsia together account for over 50% of direct maternal deaths [2]. Without access to timely, appropriate medical care, up to 63,000 women die and 500,000 pregnancies end in stillbirth as a result of preeclampsia globally each year [3]. Ninety-nine percent of these deaths are estimated to occur in resource-limited settings [3]. Preventing these outcomes requires early diagnosis, prompt referral, and skilled antenatal and delivery care including administration of magnesium sulfate. In Kenya, fewer than 30% of women attend the recommended eight antenatal visits and only approximately 60% deliver in health care facilities [3, 4]. Current efforts to reduce complications from preeclampsia/eclampsia in low and middle income countries (LMIC) have largely focused on devising clinical risk scores and creating point-of-care tests that can reliably identify women at risk of developing these disorders. Despite these interventions, one of the greatest barriers for women during pregnancy and at the time of delivery remains the cost of care [5].

Preventable pregnancy-related deaths have largely been attributed to delivery without a skilled birth attendant, generally considered a less expensive alternative to hospital-based care [6]. To address this and accelerate progress towards universal health coverage, Kenya joined other African countries in the abolishment of delivery fees in all public health facilities through a presidential directive effective June 1^st^2013 (did not include outpatient antenatal care) [6]. With little warning, public health facilities became eligible for reimbursement for every woman presenting in labour, or who gives birth during admission, through a capitation fund provided by the Ministry of Health [6]. Few facilities were prepared for the large influx of patients. Initial analysis suggests a 30% sustained increase in the number of births in public health facilities in the 24 months following policy change [6]. Similar policy changes in Ghana, Senegal, Tanzania, and Burkina Faso have all been associated with significant increases in institutional delivery rates [7, 8].

With an increase in the number of women able to access care for birth (and for the treatment of pregnancy related complications, by extension), we hypothesized that a higher proportion would be diagnosed with and treated for hypertensive disorders of pregnancy. We predicted that improved access would encourage women to present earlier in the course of their disease. This would facilitate better care and may prevent severe complications. We hypothesized that free access to magnesium sulfate would decrease the proportion of patients who develop eclampsia after presentation to hospital.

Conversely, a significant increase in the number of women presenting for care without a proportional increase in hospital resources was hypothesized to potentially overwhelm the system. This could ultimately negatively impact the care all women receive. The overall effect of free maternity services on maternal and neonatal mortality is therefore difficult to estimate. By exploring outcomes related to a specific disease, we were better able to understand perinatal care at a systems level and identify major gaps in service provision.

Without population-based tools to track health outcomes for all pregnant women in Kenya, we focused on our institution, Moi Teaching and Referral Hospital (MTRH), to assess the impact of policy change on outcomes specific to the hypertensive disorders of pregnancy. As the second-largest referral hospital in the country serving a population of over 3.5 million, MTRH provided a unique platform to conduct this study. It is often the final institution in a chain of referrals for women with pregnancy-related complications. In a study conducted at MTRH prior to this policy change, eclampsia accounted for 22% of all maternal deaths [9]. An additional 7% of all neonatal deaths were directly attributable to eclampsia and 38% to prematurity, which is often associated with severe preeclampsia [9].

## Methods

### Aim

Our primary objective was to determine the effect of free maternity care on the incidence of the hypertensive disorders of pregnancy diagnosed at a tertiary referral center in Kenya and the impact it had on related maternal and newborn outcomes. Our broader objective was to identify areas for improvement in the management of the hypertensive disorders of pregnancy at our institution and thereby decrease maternal and neonatal mortality.

### Study design

We conducted a retrospective cross-sectional chart review of women discharged or deceased with a diagnosis of gestational hypertension, preeclampsia, eclampsia or HELLP syndrome at MTRH one year before (June 1, 2012-May 31, 2013, referred to as T1) and one year after (June 1, 2013-May 31, 2014, referred to as T2) implementation of free maternity services at public facilities. We used electronic data collected by the institution to track the total number of antenatal admissions/deliveries during both time periods to compare the incidence of disease between the groups. All other data collection was by way of manual review of retrieved patient charts and recording on a hard copy, pre-designed data collection form.

### Study population

The hospital records office maintains an electronic database of discharge diagnoses for all patients. To ensure all eligible women were included, we identified those with gestational hypertension, preeclampsia, eclampsia or HELLP syndrome and cross-referenced this list with women identified as having received anti-hypertensives and/or magnesium sulfate from the hospital’s electronic pharmacy database. Based on the list generated using these criteria, hard copy patient charts were located and reviewed by two trained research assistants. *Gestational hypertension* was defined as blood pressure > / = 140/90 without proteinuria, *preeclampsia* was defined as blood pressure in the range of 140–159/90–109 and any proteinuria on urine dipstick, *severe preeclampsia* was defined as blood pressure > / = 160/110 and any proteinuria on urine dipstick, *HELLP syndrome* was defined as biochemical evidence of hemolysis, elevated liver enzymes and low platelets with or without symptoms of preeclampsia, and *eclampsia*was defined as any of the above with otherwise unexplained seizures. These definitions are based on the ACOG guideline on High Blood Pressure in Pregnancy published in 2000 and were the ones used at MTRH at the time of our chart review [10]. Current international guidelines differ in that preeclampsia is no longer defined as mild or severe, proteinuria is not required for diagnosis and HELLP syndrome is considered a manifestation of preeclampsia and not a separate entity [11]. Recent literature would suggest that any proteinuria measured on urine dipstick is predictive of worse maternal outcomes in low resource settings and its measurement remains useful for risk stratification [12].

### Data collection

Demographic information, obstetric history, medical history, details of the current pregnancy, diagnosis on admission and at discharge, antepartum treatment, maternal outcomes, and neonatal outcomes were all collected using manual chart review. Infant charts were retrieved when admission to the Newborn Unit (NBU) occurred following birth. Any patient chart in which death was recorded was reviewed independently by three members of the study team to ascertain the cause of death.

### Statistical analysis

Sample size was calculated using a 50% difference in the rate of diagnosis of eclampsia among women presenting with a hypertensive disorder in pregnancy between T1 and T2. Sample size was calculated to ensure enough patient records were retrieved to find a difference in outcomes. Based on a survey of the hospital’s database, we estimated that 10% of pregnant women at MTRH were discharged with a diagnosis of preeclampsia. This is in keeping with the estimated global prevalence of preeclampsia of 5–7% and the expected caseload at a tertiary referral hospital [1]. Using Pocock’s formula [13], we determined that we needed 432 women in each of T1 and T2 to detect a 50% difference in event rate, with 80% power and 5% significance level.

Data were entered into REDCap Version 6.10.2 and analyzed using STATA/IC 12. The data were grouped into two periods spanning the year before the introduction of free maternity services (June 1, 2012-May 31, 2013) and the year after its introduction (June 1, 2013-May 31, 2014) and presented in tables comparing the groups (labeled T1 and T2). Descriptive data including means for continuous variables and proportions for categorical variables were compared using t-test and chi-square tests, respectively with *p* < 0.05 considered significant. When the expected frequency was less than 5, Fisher’s exact *p*-value was used instead of the chi-square *p*-value.

## Results

**Fig. 1:**
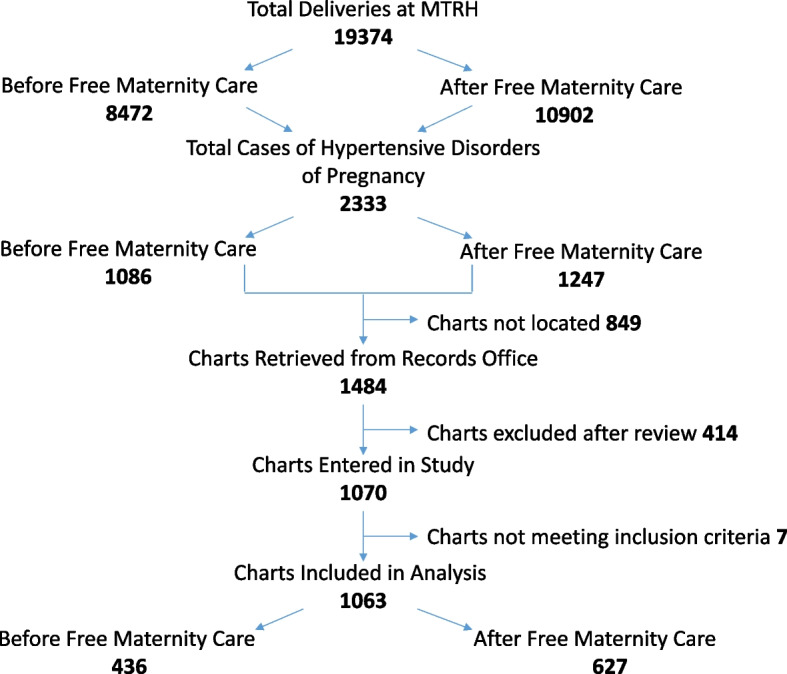
Chart selection map – Total number of deliveries at MTRH during the 2 year study period (June 1, 2012 to May 31, 2014). 414 charts were excluded after review because the diagnosis was incorrect. There were a similar proportion of missing charts in both groups (650 charts before and 620 charts after introduction of free maternity services missing). Missing charts/patient data were, unfortunately, likely a consequence of poor record keeping; selection bias was not suspected because demographic data and outcomes were similar between the groups (see below)

There were a total of 19,374 births at MTRH between June 1, 2012 and May 31, 2014. There were 8,472 births in T1 and 10,902 births in T2. The proportion of women diagnosed with a hypertensive disorder of pregnancy was higher in T1 (12.8% vs 11.4%, *p* = 0.0029). Overall, 60% of the hard copy charts belonging to women with a hypertensive disorder of pregnancy were available for data collection. Our analysis included 436 patients in T1, and 627 in T2 (similar proportion of available charts for data collection in each group). Please refer to Fig. 1 for further details.

As shown in Table 1, demographic characteristics were similar in T1 and T2. There was no difference in distance traveled to hospital or in the referral rate from smaller, community facilities. There was no difference in the proportion of women with a history of chronic hypertension or diagnosis of preeclampsia or eclampsia in a previous pregnancy.

**Table 1:** Background demographic and health characteristics

Demographic	Before (T1) (*n* = 428)	After (T2) (*n* = 623)	*P* value
**Age (mean)**	27.4 (SD 6.06)	27.4 (SD 6.06)	0.945
Age < 20	32 (7.5%)	38 (6.1%)	0.379
Age > 35	47 (11.0%)	67 (10.8%)	0.907
**Parity**			
0	216 (49.5%)	311 (49.6%)	1.000
1–4	179 (41.1%)	257 (41.0%)	
4 +	41(9.4%)	59 (9.4%)	
**Distance from Hospital (km)**	23.6 (SD 37.6)	25.6 (SD 37.1)	0.415
**Referred from Periphery**	154 (40.3%)	242 (42.6%)	0.481
**GA (at admission to MTRH)**			
< 28 weeks	17 (4.6%)	28 (5.3%)	0.619
28–34	61 (16.4%)	107 (20.2%)	0.151
> 34 weeks	293 (79.0%)	393 (74.5%)	0.118
**Education**			
None	2 (0.5%)	3 (0.5%)	0.744^f^
Primary	40 (9.2%)	53 (8.5%)	
Secondary + Post-Secondary	148 (34%)	230 (36.7%)	
Missing	246 (56.3%)	341 (54.3%)	
**Marital status**			
Single	78 (17.8%)	112 (17.9%)	0.425^f^
Married	351 (80.5%)	505 (80.5%)	
Separated/Divorced	2 (0.5%)	0 (0.0%)	
Missing	5 (1.2%)	10(1.6%)	
**Medical History**			
cHTN	15(0.03%)	25(0.04%)	0.389
RHD	1 (0.0%%)	0 (0.0%)	0.230
Diabetes	1 (0.2%)	2 (0.3%)	1.000^f^
Renal disease	2 (0.5%)	0 (0.0%)	0.168^f^
Pulmonary disease	7 (1.6%)	6 (1.0%)	0.344
Neurologic disease	6 (1.4%)	3 (0.5%)	0.172^f^
HIV	17 (3.9%)	27 (4.3%)	0.743
**History of**			
PET	22 (9.4%)	25 (7.3%)	0.367
Eclampsia	4 (0.92%)	2 (0.32%)	0.803^f^

All background characteristics included for evaluation were similar between groups

*GA* Gestational age, *cHTN* Chronic hypertension, *RHD* Rheumatic heart disease, *HIV* Human Immunodeficiency Virus, *PET* Preeclampsia

^f^ Fisher’s exact

As illustrated in Table 2, there was no change in the proportion of women delivering live births. Nearly all women delivered at MTRH (96%) (as opposed to a peripheral facility, before referral or in transit). There was a trend towards increased preterm delivery but this did not reach statistical significance. There was no change in the median time from admission to delivery (1 day).

**Table 2:** Admission and delivery outcomes among women with live births

	**Before (T1) (*n* = 437)**	**After (T2) (*n* = 626)**	***P* value**
Live births	394 (90.2%)	551 (88.0%)	0.274
Stillbirths^a^	43 (9.8%)	75 (12%)	0.391
**GA (at delivery) mean**			
< 28 weeks	4 (1.2%)	6 (1.3%)	0.928
28–34 weeks	43 (13.0%)	77 (16.5%)	0.179
> 34 weeks	280 (84.9%)	467 (80.9%)	0.153
**Median Interval time from admission to delivery, range (days)**	1 (0—32) Mean = 2.1 ± 4.6	1 (0—46) Mean = 2.2 ± 4.6	0.240 0.754
**Location of delivery**			
MTRH	367 (96.8%)	518 (95.9%)	0.578^f^
Hospital (other then MTRH)	8 (2.1%)	18 (3.3%)	
Home/Transit)	4 (1.1%)	5 (5.3%)	
**Mode of Birth**			
SVD	239 (63.1%)	347 (64.1%)	0.218^f^
Assisted VD	6 (1.6%)	8 (1.5%)	
CS	134 (35.3%)	182 (33.6%)	
Not recorded	0 (0.0%)	4 (0.7%)	
**Plurality**			
Singleton	364 (92.9%)	520 (94.5%)	0.566^f^
Twins	27 (6.9%)	28(5.1%)	
Triplets	0 (0.0%)	1 (1.2%)	
**Birth weight**			
	2750.9 (700–4350)	2685.6 (270–4850)	0.03
	Mean 2652 ± 702.7	Mean 2568 ± 769.6	0.096
**Maternal median and range laboratory values during admission**			
Hemoglobin (mg/dl)	13.3 (3.0 – 17.1)	11.6 (3.2–19.6)	0.369^ m^
Platelets	201.5 (13–640)	186 (6–618)	0.034 ^m^
ALT (IU)	14.3 (0.4–2,362)	15.2 (1.9–1,399.5)	0.102 ^m^
AST(IU)	25.7 (10.1–3,112.8)	27.2 (6.0–3,977.4)	0.403 ^m^
Creatinine (mg/dL)	58 (4.4–873)	66 (1.4–1,669)	< 0.001 ^m^
**Maternal median and range BP recorded during admission**			
Systolic (mmHg)	148.5 (109.5–221)	150 (119.5–205)	0.017 ^m^
Diastolic (mmHg)	95 (39.5–160)	98 (48.5 – 160)	< 0.001^ m^

*SVD* Spontaneous vaginal delivery*, assisted VD* assisted vaginal delivery *(with Kiwi vacuum), CS* Cesarean section

^a^ Due to facility record-keeping standards, detailed delivery information about stillbirth cases was not uniformly available and therefore these cases were not included for analysis here (see Table 5)

^f^ Fisher’s exact

^m^ Median test

### Scope of hypertensive disorders

Table 3 outlines the distribution of hypertensive disorders in T1 and T2. Among those women diagnosed with a hypertensive disorder of pregnancy, there was an upward trend in the proportion of women diagnosed with eclampsia and HELLP in T2, although this did not reach statistical significance. There was a statistically significant increase in the proportion of women diagnosed with gestational hypertension (4.1% vs 8.1%, *p* = 0.009).

**Table 3:** Distribution of Hypertensive Disorders (as previously defined – see Background)

Hypertensive disorders	Before (T1) (*n* = 360)	After (T2) (*n* = 529)	*P* value
gHTN	18 (4.1%)	51 (8.1%)	0.009
PET	199 (45.6%)	266 (42.4%)	0.298
Severe PET	95 (21.8%)	114 (18.2%)	0.146
Eclampsia	33 (7.6%)	63 (10.1%)	0.165
HELLP	15 (3.4%)	35 (5.6%)	0.105

*gHTN* Gestational hypertension*, PET* Preeclampsia*, HELLP* Hemolysis, elevated liver enzymes, low platelets

There was a statistically significant decrease in the number of women who presented to hospital for concerns specific to labor and delivery (labor pains and/or suspected rupture of membranes) (54.4% vs 45.6%, *p* = 0.015 and 11.7% vs 6.5%, *p* = 0.005, respectively) in T2, as illustrated in Table 4. Many women presented for more than one reason in both T1 and T2.

**Table 4:** Reason for Presentation to MTRH

Reason for Presentation of Women Diagnosed with Hypertensive Disorder of Pregnancy	Before (T1) (*n* = 436)	After (T2) (*n* = 627)	*P* value
Lower abdominal pains/Labor	237 (54.4%)	286 (45.6%)	0.015
Drainage of liquor/SROM	51 (11.7%)	41 (6.5%)	0.005
Headache	131 (30.0%)	216 (34.5%)	0.314
Swelling/Edema	123 (28.3%)	177 (28.3%)	0.965
Epigastric/RUQ pain	71 (16.3%)	82 (13.1%)	0.121
Decreased Fetal movement	44 (10.1%)	62 (9.9%)	0.659
Visual changes	43 (9.9%)	56 (8.9%)	0.817
Nausea ± vomiting	20 (4.6%)	34 (5.4%)	0.799

*SROM* Spontaneous rupture of membranes*, RUQ* Right upper quadrant

### Adverse outcomes

Among those women diagnosed with hypertensive disorders, there was no difference in the proportion who developed obstetric or medical complications between T1 and T2. There was a statistically significant increase in the proportion of women who died as a result of their condition (1.2% vs 3.03%, *p* = 0.042).

There was a significantly increased incidence of preterm births between 28–34 weeks in T2 when stillbirths were included in the analysis (13.5% vs 21.6%, *p* = 0.003). There were also more neonates with Apgar scores less than 7 at 1 min (23% vs 29%, *p* = 0.048). There was a trend towards higher admission rates to the NBU, stillbirths, and neonatal deaths, although these differences did not reach statistical significance. There was no difference in fetal/neonatal adverse outcomes among women with multiple gestation (data not shown). Please refer to Table 5 for full details of maternal and neonatal outcomes.

**Table 5:** Significant maternal and neonatal outcomes in women with hypertensive disorders of pregnancy

Severe Maternal Outcomes	Before (T1) (*n* = 436)	After (T2) (*n* = 627)	*P* value
**Death**	**5 (1.15%)**	**19 (3.03%)**	**0.042**
Postpartum Hemorrhage^a^	58 (13.3%)	76 (12.1%)	0.568
Placental abruption	10 (2.3%)	6 (1.0%)	0.078
Intensive Care Unit admission	8 (1.8%)	8 (1.3%)	0.462
Disseminated intravascular coagulation	1 (0.23%)	7 (1.12%)	0.151^f^
Blood transfusion	45 (10.3%)	85 (13.6%)	0.113
Seizure/convulsion	33 (7.6%)	63 (10.05%)	0.165
Need for dialysis	4 (0.92%)	13 (2.07%)	0.139
VTE	5 (1.15%)	2 (0.32%)	0.130^f^
Myocardial Infarction	2 (0.5%)	1 (0.2%)	0.571^f^
Pulmonary Edema	3 (0.69%)	7 (1.1%)	0.539^f^
Stroke	3 (0.69%)	4 (0.64%)	1.000^f^
Platelets < 50	9 (2.3%)	25 (4.5%)	0.073
Transaminitis (ALT or AST > 70 IU)	55 (14.2%)	102 (19.6%)	0.034
**Total with an adverse maternal outcome**	147(33.6%)	226(36.1%)	0.408
**Neonatal Outcomes**			
Delivery at < 28 completed weeks	12 (3.5%)	13 (2.6%)	0.434
Delivery at 28–34 completed weeks	46 (13.5%)	108 (21.6%)	0.003
Delivery at 34–37 completed weeks	55 (16.2%)	91 (18.2%)	0.455
Low Birth Weight (< 2.5 kg)	141 (38.0%)	237 (42.8%)	0.148
NBU Admission	107 (26.4%)	189 (32.1%)	0.052
IUFD/Stillbirth	41 (10.1%)	70 (11.9%)	0.391
Neonatal Death	12 (5.2%)	28 (4.8%)	0.160
**Total with adverse fetal/neonatal outcomes**	224(55.2%)	353 (59.9%)	0.135

*VTE* Venous thromboembolism, *NBU* Newborn Unit, *IUFD* Intra-uterine fetal demise

^a^ Postpartum hemorrhage defined as > 500 mL after vaginal delivery and > 1000 mL after cesarean section

^f^ Fisher’s exact

### Pharmacological management

As demonstrated in Table 6, there was a statistically significant increase in the use of magnesium sulfate for seizure prophylaxis (77.3% vs 84.5%, *p* = 0.003), mostly among women with gHTN (20.2% vs 24%, *p* = 0.020). There was a trend towards less use of magnesium sulfate among women with eclampsia and HELLP syndrome. There was no overall difference in the use of anti-hypertensives between groups. Less than 50% of women between 28–34 weeks received dexamethasone for fetal lung maturity in both groups.

**Table 6:** Pharmacological management of hypertensive disorders of pregnancy

Medication	Before (T1) (*n* = 436)	After (T2) (*n* = 627)	*P* value
**Magnesium sulfate**	337 (77.3%)	530 (84.5%)	0.003
gHTN	7 (30.4%)	24 (42.1%)	0.02
PET	147 (73.9%)	231 (86.8%)	0.298
Severe PET	81 (85.3%)	107 (93.9%)	0.146
Eclampsia	33 (100%)	58 (92.1%)	0.165
HELLP	14 (93.3%)	31 (88.6%)	0.105
**Anti-Hypertensives**	433 (99.3%)	618 (98.6%)	0.378^f^
Nifedipine (oral)	374 (85.8%)	563 (89.8%)	0.046
Labetalol (IV)	80 (18.4%)	142 (22.6%)	0.09
Hydralazine (IV and/or oral)	27 (6.2%)	26 (4.2%)	0.132
Methyldopa (oral)	24 (5.5%)	27 (4.3%)	0.369
Atenolol (oral)	13 (3.0%)	40 (6.4%)	0.012
**Dexamethasone**			
< 28 weeks	4 (30.9%)	5 (29.4%)	0.929
28–34 weeks	24 (44.4%)	54 (48.2%)	0.645
34–37 weeks	13 (22.4%)	24 (34.8%)	0.008
> 37 weeks	11 (4.7%)	5 (1.7%)	0.045

Medications unrelated to the direct management of a patient’s hypertensive disorder were not included in the analysis. Only those patients receiving the correct dose have been included as having received a medication. Of note, oral labetalol is not used at MTRH

^f^Fisher's exact

## Discussion

This study aimed to evaluate the impact of free maternity services on maternal and neonatal outcomes related to the hypertensive disorders of pregnancy at a tertiary referral center in western Kenya. There was a significant increase in the number of women presenting to MTRH for delivery after policy change, in keeping with statistics available from other public facilities across the country [5, 6]. There was no change in the proportion of women being referred from smaller, community facilities and no change in the mean distance traveled to receive care. There was no change in the proportion of women diagnosed with eclampsia or HELLP syndrome after policy change but there was a significant increase in the proportion of women diagnosed with gestational hypertension. Critically ill women were referred for tertiary level expertise at the same rate, regardless of the cost of care. The increased case rate of mild gestational hypertension was likely because the diagnosis was an incidental finding when women presented in labor.

Significantly fewer women with hypertensive disorders presented with symptoms related to labor (labor pains and/or suspected rupture of membranes) after policy change. It may be inferred that free maternity services are encouraging women with warning signs to present for antenatal care at a higher level facility earlier in the course of their disease/before labor. There was, however, no improvement in the proportion of women who developed serious complications from their condition. Perhaps the most striking finding in this study was that significantly more women died in hospital of their condition after policy change. This may indicate that women who would have otherwise died at home presented to hospital for care or may suggest that this tertiary care facility was not able to manage the influx of patients after free maternity services were introduced.

There was a significant increase in the proportion of preterm births between 28–34 weeks when stillbirth was included in our analysis, but no change in length of admission before delivery. This may indicate an increase in very sick women presenting at earlier gestations requiring delivery, increased recognition of fetal distress, or an appropriate increase in iatrogenic preterm delivery secondary to improved management of severe maternal disease. There was a trend towards increased admission rate to the NBU, stillbirth, and neonatal death after the policy change but these did not reach statistical significance.

After policy change, more women with gestational hypertension were treated with magnesium sulfate prophylaxis. There was a trend towards less use of magnesium sulfate in women with severe preeclampsia, HELLP syndrome and eclampsia, although this did not reach statistical significance. There was no difference in the use of anti-hypertensives between the groups. Less than 50% of women received steroids for fetal lung maturity; this did not improve after policy change. To our knowledge, interrupted drug supply did not account for these observations. These findings indicate that without adequate supplies or training among health care workers, and with a large increase in patient volumes, appropriate management of pregnant women with hypertensive disorders has suffered and was poor to begin with.

Ours is one of very few studies to evaluate the impact of a demand side financing strategy on outcomes related to a specific maternal diagnosis in LMIC. Preeclampsia is responsible for 1 in 7 maternal deaths worldwide and 1 in 4 perinatal deaths [14]. It is a major contributor to maternal and neonatal mortality and is worthy of independent investigation to identify gaps in service provision and areas for improvement. Ours is also the only study evaluating the impact of a free maternity services policy on the use of essential medications like magnesium sulfate, anti-hypertensives, and steroids for fetal lung maturity in LMIC.

Data collection and analysis suffered from a number of limitations. Generalizability of our findings at the national and international level may not be possible given that we sampled only a tertiary referral center in western Kenya. Our cohort may not capture the dynamics of regional factors affecting other health facility management of preeclampsia. A prospective analysis was not possible before policy change because it was announced with little warning and implemented immediately. Therefore, only retrospective analysis could be performed. With more time and resources, more extensive analysis over several years may have revealed subtle trends our study was not powered to detect. As is the case with many chart reviews in LMIC, there was a significant proportion of missing files and incomplete patient data. Although it is unlikely the missing data was biased and changed the outcomes we observed, we cannot be certain. It was not possible to ascertain when patients made the decision to seek medical attention during the course of their disease. This information may have shed light on outcomes that could not have been mitigated or prevented by adequate management on arrival due to very late presentation.

Over the last three decades, governments of LMIC have implemented a variety of strategies to incentivize pregnant women to seek more timely and appropriate care in order to reduce maternal and infant mortality. Evaluations of the impact of user fee removal, including nationwide government policy, have generally found evidence of increased hospital-based delivery rates [13, 15, 16]. This is in keeping with our findings and those of others who have reviewed the free maternity services policy in Kenya over the past eight years [5, 6]. Unfortunately, analysis has shown no overall change in the maternal or neonatal mortality rates in Kenya to date [6, 17]. This is also in keeping with our findings specific to outcomes related to the hypertensive disorders of pregnancy and those of studies examining other related policy changes in Kenya [18–22]. In Nigeria, despite adoption of free maternal and child healthcare policies, mortality has remained higher than expected 15 years later [22, 23]. Kenya may be following in the same footsteps if further action isn’t taken.

Although a necessary measure, policy change alone does not address many of the other barriers women in LMIC face to access pregnancy-related health care. This study informs policy makers that providing free maternity services does, indeed, improve hospital delivery rates, which should be considered a major success. More is needed, however. Free maternity services do not account for the cost of seeking care (transportation) nor the lost opportunity costs related to both seeking and receiving care (lost income generating opportunities). Cost is not the only factor preventing women from utilizing health facilities. In Kenya, preventable maternal and neonatal deaths have also been attributed to the need to travel long distances to reach health centers and cultural practices that encourage women to deliver at home with traditional birth attendants [6].

Poor quality of care at facilities remains a major contributing factor to high maternal and neonatal mortality rates in LMIC. This is evident in our study and should be the focus of future efforts to help inform policy makers and medical staff on areas of improvement at the health systems level [23]. In Nigeria, identified limitations to care provision have included irregular and interrupted drug supply, health worker absenteeism, poor supervision and monitoring, weak referral systems, absence of written care guidelines, staffing shortages, inconsistent recording and reporting, poorly motivated health workers, mistrust between patients and providers, and inadequate physical infrastructure [23–25]. The majority of these limitations have been described in Kenya following the policy change in 2013 and are highlighted by the lack of improved outcomes observed in our study [6]. Targeted initiatives addressing individual challenges with measureable outcomes should be the mainstay of future innovations.

In 2013, a service readiness availability map was conducted in Kenya when the policy change was implemented. Results demonstrated that only 28% of facilities in the country had essential medicines for pregnancy-related emergencies, including magnesium sulfate [1, 26]. This demonstrates the inability of public health facilities to adequately manage even the basic principles of preeclampsia care and reflects our findings. Reliable access to life saving medication and equipment is evidently a major barrier impeding improvement to maternal and neonatal outcomes that must be urgently addressed.

Initiatives focusing on risk stratification tools and early identification of women at risk of developing preeclampsia in LMIC often fail to consider the myriad of barriers women face after diagnosis. The data presented here suggest that no one strategy targeting only one barrier faced by such women will be sufficient to affect change. Failure to measure improved maternal or neonatal outcomes does not necessarily indicate that an intervention has failed, but instead illuminates other service gaps to be addressed. At MTRH, a Maternal Fetal Medicine clinical fellowship was initiated in 2019, in collaboration with the University of Toronto, to help address a number of the facility-based factors identified after policy change. Training in the fellowship strongly emphasizes patient and family education and counseling. The added local health provider training, education and supervision are hoped to have a sustainable positive impact on patient care. Data collection remains ongoing. The hospital has also recently approved the implementation of a standardized order set for the inpatient antenatal management of the hypertensive disorders of pregnancy as a means to address potential gaps in provider knowledge and to streamline access to necessary care. Strategies to improve early screening and disease detection on an outpatient basis are also being explored in the newly initiated High Risk Antenatal clinic.

## Conclusion

In summary, this study demonstrates that more women presented to a tertiary care facility for labor and delivery in western Kenya after the introduction of free maternity services. There was no increase in the proportion of women who presented with hypertensive disorders of pregnancy and no improvement in related outcomes. There was also no improvement in the use of essential medications. This demonstrates that in isolation, free maternity services, however necessary, are insufficient to improve preeclampsia related outcomes in Kenya. Multiple complementary strategies acting in unison are urgently needed.

## Data Availability

The raw dataset used during the current study is available from the corresponding author on reasonable request. All data analysed are included in this published article.

## Abbreviations

ACOG: American College of Obstetricians and Gynecologists
Assisted VD: Assisted Vaginal Delivery
cHTN: Chronic Hypertension
CS: Cesarean Section
DIC: Disseminated Intravascular Cooagulopathy
GA: Gestational age
gHTN: Gestational Hypertension
HELLP: Hemoloysis, Elevated Liver Enzymes, Low Platelets
HIV: Human Immunodeficiency Virus
IUFD: Intra-uterine Fetal Demise
LMIC: Low and Middle Income Countries
MTRH: Moi Teaching and Referral Hospital
NBU: Newborn Unit
PET: Preeclampsia
RHD: Rheumatic Heart Disease
RUQ: Right upper quadrant
SROM: Spontaneous Rupture of Membranes
SVD: Spontaneous Vaginal Delivery
VTE: Venous Thromboembolism
